# Energy expenditure during overfeeding

**DOI:** 10.1186/1743-7075-3-25

**Published:** 2006-07-12

**Authors:** Annemiek MCP Joosen, Klaas R Westerterp

**Affiliations:** 1 Department of Human Biology, Maastricht University, P.O. Box 616, 6200 MD Maastricht, The Netherlands

## Abstract

The large inter-individual variation in weight gain during standardized overfeeding together with a weight gain that is often less than theoretically calculated from the energy excess suggest that there are differences between persons in the capacity to regulate energy expenditure and hence metabolic efficiency. Adaptive thermogenesis is defined as the regulated production of heat in response to environmental changes in temperature and diet, resulting in metabolic inefficiency. The question is whether adaptive thermogenesis can be identified in overfeeding experiments. From the numerous human overfeeding experiments we selected those studies that applied suitable protocols and measurement techniques. Five studies claimed to have found evidence for adaptive thermogenesis based on weight gains smaller than expected or unaccounted increases in thermogenesis above obligatory costs. Results from the other 11 studies suggest there is no adaptive thermogenesis as weight gains were proportional to the amount of overfeeding and the increased thermogenesis was associated with theoretical costs of an increased body size and a larger food intake. These results show that in humans, evidence for adaptive thermogenesis is still inconsistent. However, they do not rule out the existence, but emphasize that if present, adaptive changes in energy expenditure may be too small to measure considering measurement errors, errors in assumptions made and small (day-to-day) differences in physical activity. In addition, it is not clear in which component or components of total energy expenditure adaptive changes can occur and whether components can overlap due to measurement limitations.

## Introduction

Obesity develops when energy intake (EI) exceeds energy expenditure (EE) for longer periods. However, overfeeding experiments show that weight gain is often less than expected from the energy excess. In part this is the result of an obligatory increase in EE associated with the increased body weight and fat-free mass [1] and the larger amount of food to be digested and absorbed [2]. In addition, there is a wide inter-individual variation in weight gain on the same amount of overfeeding, which suggests that some persons can regulate their EE beyond the obligatory costs associated with weight gain to resist weight gain.

EE consists of obligatory EE required for the normal functioning of cells and organs, EE for physical activity, and adaptive (or facultative) thermogenesis, which is defined as the regulated production of heat in response to environmental changes in temperature and diet [3]. The obligatory EE is generally calculated from theoretical values based on body weight, body composition and energy intake [4]. The EE for physical activity can be directly measured from the work performed on the environment. In contrast, though the definition is clear, the determination of adaptive thermogenesis merely depends on changes in EE that are unaccounted for changes in obligatory EE. Adaptive thermogenesis then reflects changes in metabolic efficiency [5].

For heat production to increase in response to environmental factors like diet the coupling between mitochondrial oxidation and ATP synthesis must be reduced. Alternatively, the use of ATP can be increased without functional results, which can be referred to as wasting of ATP futile cycles [3]. Differences in the capacity for adaptive changes in thermogenesis may be involved in the efficiency of weight gain and hence a predisposition to obesity. However, the relevance of adaptive thermogenesis in the etiology of obesity is controversial, as adaptive changes in EE are believed to be no more than a few percent [6-8]. But as obesity is the result of an EI that exceeds EE for longer periods, even a slightly positive energy balance (EB) on a daily basis can lead to a significant weight gain over years. The question is whether there is experimental evidence for adaptive thermogenesis as a mechanism to resist weight gain. For this purpose we selected 16 human overfeeding experiments based on their protocols (i.e. the amount and duration of overfeeding) and techniques used to measure EE. In addition, we only selected studies with healthy adult subjects, with a weight maintenance baseline period directly before overfeeding and information on energy expenditure and weight gain.

### Human overfeeding experiments

Obesity needs a positive energy balance to develop, a situation that is mimicked in overfeeding experiments. Our selection of human overfeeding studies is summarized in Table 1. To investigate the importance of adaptive thermogenesis, the component or components of energy expenditure involved need to be defined and reflected in the study design.

**Table 1:** Selection of human overfeeding experiments

Reference	Subjects	Overfeeding period/setting	Dietary intake*	EE measurements	Limited activity	Mean weight gain	Changes in EE**	Costs of weight gain (MJ/kg)***	Adaptive thermogenesis?
Bouchard et al./Tremblay et al. [22, 30]	24 males (12 twin pairs) normal weight	84 d metabolic unit	+ 4.2 MJ/d 15% P, 35% F, 50% CHO	RMR, DIT	yes	8.1 ± 2.4 kg	ΔRMR 0.69 ± 0.60 MJ/d	44	no
Dallosso and James [41]	8 males normal weight	7 d metabolic unit	150% base 50% F	TEE, SMR, BMR	low or high exercise	1.2 ± 0.5 kg	ΔTEE 5.6% (low), 6.4% (high)	39	yes
Diaz et al. [4]	6 males normal/overweight	42 d metabolic unit	150% base 12% P, 42% F, 46% CHO	ADMR, TEE, BMR	no	7.6 ± 1.6 kg	ΔBMR 0.9 ± 0.4, ΔDIT + AEE 0.9 ± 2.1 MJ/d	35	no
Forbes et al. [23]	2 males, 13 females normal weight	17–21 d metabolic unit	total 79–159 MJ; 15% P, 45–50% F, 45–50% CHO	BMR	no	4.4 ± 0.6 kg	ΔBMR 0.49 ± 0.46 MJ/d	28	no
Glick et al. [40]	8 females normal/overweight	5 d metabolic unit	+ 9.5 MJ/d 13% P, 38% F, 50% CHO	O_2_-consump. during rest and exercise	yes	1.8 ± 0.3 kg	no	26	no
Horton et al. [14]	16 males normal weight/obese	14 d	+ 50% base entirely F or CHO	TEE	no	2.7 kg	ΔTEE 0.9 (CHO) MJ/d	CHO 90 F 100	no
Jebb et al. [24]	3 males normal weight	12 d respiration chamber	133% base 15% P, 35% F, 50% CHO	TEE, BMR	yes	2.9 kg	ΔBMR 0.42 MJ/d ΔTEE 0.75 MJ/d		no
Joosen et al. [19]	14 females normal weight	14 d outpatients	150% base 7% P, 40% F, 53% CHO	ADMR, BMR, PA	no	1.5 ± 0.9 kg	ΔBMR 0.38 ± 0.47 MJ/d	54	no
Lammert et al. [20]	20 males normal weight	21 d metabolic unit	+ 5 MJ/d high F or high CHO	SMR	pairs according to habitual PA	1.5 kg	no	CHO 87 F 64	no
Levine et al. [25]	12 males, 4 females normal weight	56 d outpatients	+ 4.2 MJ/d 20% P, 40% F, 40% CHO	ADMR, BMR, DIT, NEAT, PA	volitional exercise constant and low	4.7 ± 1.8 kg	ΔBMR 0.33 ± 0.53 ΔDIT 0.58 ± 0.35, ΔNEAT 1.38 ± 1.08 MJ/d	50	yes
Norgan and Durnin [36]	6 males normal weight	42 d metabolic unit	+ 6.2 MJ/d 12% P, 33% F, 38% CHO, 7% alcohol	resting and mobile activities	sedentary (leisure) activities	6.0 ± 1.8 kg	no	43	no
Pasquet et al. [26]	9 males normal weight	61–65 d 'Guru Walla'	total 955 ± 252 MJ; 15% P, 15% F, 70% CHO	ADMR, RMR, ppRMR, PA	no	17 ± 4 kg	ΔRMR 44 ± 10, ΔppRMR 26 ± 12, ΔPA -40 ± 21%	56	yes
Ravussin et al. [27]	5 males normal weight	9 d outpatients	160% base 15% P, 39% F, 46% CHO	TEE, BMR, DIT, PA	-	3.2 ± 0.3 kg	ΔSMR 1.05, ΔBMR 0.62, ΔDIT 0.58 MJ/d	23	no
Roberts et al. [28]	7 males normal weight	21 d outpatients	+ 4.2 MJ/d 10% P, 40% F, 50% CHO, 0.2% alcohol	ADMR, RMR, DIT, PA	no	2.5 ± 3 kg	ΔRMR 0.63 ± 0.20 MJ/d	36	no
Webb and Annis [21]	6 males, 6 females normal/overweight	30 d outpatients	+ 4.2 MJ/d high P+F, high CHO or average	TEE	yes	2.7 kg (average, CHO) 1.8 kg (P+F)	ΔTEE 7.4%	P+F 72 CHO 46 average 47	yes
Zed and James [29]	16 females normal weight/obese	6 d metabolic unit	+ 4.3 MJ/d entirely F	TEE, SMR, BMR, DIT	-	1 kg	ΔBMR 9.4% (lean)	78 (normal weight only)	yes

ADMR = average daily metabolic rate, BMR = basal metabolic rate, DIT = dietary-induced thermogenesis, CHO = carbohydrate, F = fat, P = protein, NEAT = nonexercise activity thermogenesis (ADMR-BMR-DIT), PA = physical activity, ppRMR = prostprandial resting metabolic rate (RMR+DIT), RMR = resting metabolic rate, SMR = sleeping metabolic rate, TEE = total energy expenditure.

* % macronutrient = energy percentage.

** statistically significant changes only.

*** calculated as mean excess energy intake divided by mean body weight gain (note: see discussion)

The most reliable studies with regard to overfeeding are studies conducted with subjects living in the research institute during the entire study period. Then, however, a disadvantage is the induction of different lifestyles even when physical activity is not limited. In other experiments, subjects are studied as outpatients, who consume one or more meals per day at the research institute, but otherwise stay in their own environments. In both conditions, the gold standard for measuring EE over longer periods is the doubly labeled water (DLW) method. In combination with sleeping or basal metabolic rate (SMR or BMR), activity-induced energy expenditure (AEE) can be determined without restricting the subjects. In addition, physical activity (PA) can be objectively measured with accelerometers, which measure body movements in terms of frequency, duration and intensity [9]. Respiration chambers allow measurements of total energy expenditure (TEE) and its components (SMR, BMR, diet-induced thermogenesis (DIT) and AEE). Though PA is limited due to the confined area, there is still considerable variation between subjects while intra-individual variation is low [2].

The duration of the overfeeding mainly determines the reliability of changes in body weight as a reflection of energy storage. The overfeeding period should be long enough to expect an increase in body weight in excess of changes due to bowel contents and edema (i.e. excessive storage of body fluids that is not the result of an increased lean body mass).

### Energy expenditure during overfeeding

#### Macronutrient intake and oxidation

When digested foods enter the bloodstream there is an oxidative hierarchy. The macronutrient that is most easily stored (fat) is oxidized last, while macronutrients that can not be stored at all (alcohol), or that can only be stored under certain circumstances (protein) or in limited amounts (carbohydrate) are oxidized first [10]. Alcohol ingestion directly increases alcohol oxidation, which is maintained until all alcohol is cleared. Protein and carbohydrate oxidation closely follow intake. In contrast, fat intake does not stimulate fat oxidation. Moreover, fat oxidation is inhibited by high intakes of the other macronutrients [10-12]. The thermic effect of the separate macronutrients is 20 to 30% for protein, 5 to 15% for carbohydrate, and 0–3% for fat [12]. The figure for the thermic effect of alcohol is not clear, values range between 6 and 30% in different studies [13].

The intake of any macronutrient in excess of energy needs will lead to fat storage, but a reduced capacity for fat oxidation could particularly predispose to obesity. Diaz et al. [4] overfed subjects 50% above baseline energy requirements for 42 d, which suppressed fat oxidation by 37% in lean subjects, but by 64% in overweight subjects. These results were confirmed by Horton et al. [14] who overfed 50% above baseline energy intake with isoenergetic amounts of fat and carbohydrate for 14 d. During both overfeeding periods, obese subjects had a higher average RQ and oxidized proportionally more carbohydrate than lean subjects. However, EE increased proportionally with the increased body size and tissue gain leaving no evidence for adaptive thermogenesis. The capacity for fat oxidation, therefore, does not seem to relate to the capacity for adaptive thermogenesis.

DIT is increased on a high-protein, high-carbohydrate diet compared to a high-fat diet [11]. In contrast, low-protein diets result in increased DIT as well. This apparent contradiction is attributed to a mechanism for enriching nutrient-deficient diets while dissipating the excess energy on low-protein diets, whereas high-protein diets result in increased thermogenesis due to the high cost of metabolizing protein [15,16]. In this context it is important to note that the term DIT is not only used for the increase in EE above BMR during the first hours after a meal, but also includes adaptive changes in BMR in response to the diet. Dulloo and Jacquet [16] reviewed the results of the normal- (15 energy%) and low- (3 energy%) protein overfeeding of Miller [17,18] who overfed five young adults of normal body weight with 4.2 MJ/d or more for 3–6 weeks. The energy costs of weight gain on the low-protein diet (80 to >300 MJ/kg body weight) were much higher than on the high-protein diet (25–45 MJ/kg body weight) suggesting that low-protein overfeeding induces adaptive changes in EE. They concluded that the capacity for adaptive thermogenesis is individually determined, as the energy costs of weight gain on normal- and low-protein overfeeding were positively related. Therefore, Stock [15] and Dulloo and Jacquet [16] suggested low-protein overfeeding as a tool to discriminate between metabolically efficient and metabolically inefficient persons by maximizing differences in thermogenesis. However, we overfed healthy females 50% above baseline energy requirements for 14 d with a low-protein (7 energy%) diet (Table 1) and did not find adaptive changes in energy expenditure [19].

The limited storage capacity for carbohydrates forces an increase in carbohydrate oxidation with carbohydrate overfeeding, which together with a decrease in fat oxidation results in a positive fat balance [14]. However, the influence of the carbohydrate content of the (overfeeding) diet on metabolic efficiency is less clear. Though not always intentionally, overfeeding diets are generally high in carbohydrates. The effects of carbohydrates are thus only comparable between diets supplying the energy excess entirely as fat (or protein) or as carbohydrates, or respectively relatively low- and high-carbohydrate diets (Table 1; refs: [14,20,21]). Lammert et al [20] overfed subjects a high-fat (energy percentages from protein:fat:carbohydrate were 11:58:31) or a high-carbohydrate diet (en% P:F:CHO 11:11:78). Calculated from the mean overfeeding of 118 (high-CHO) and 101 MJ (high-F) and the mean weight gains of 1.35 (high-CHO) and 1.58 kg (high-F), the costs of weight gain were 87 and 63 MJ/kg respectively. In contrast, the cost of weight gain on a high-protein/high-fat diet (en% P:F:CHO 20:50:30) were 72 MJ/kg compared to ~47 MJ/kg on both an average (en% P:F:CHO 14:41:45) and a high-carbohydrate (en% P:F:CHO 10:30:60) in the study of Webb and Annis [21]. Results from the study of Horton et al [14] appear to point towards the same direction with costs of weight gain 100 MJ/kg on high-fat and 90 MJ/kg on high-carbohydrate overfeeding. While the first study suggests that costs of weight gain are increased with high-carbohydrate overfeeding which might be caused by *de novo *lipogenesis, the last two studies suggest that costs of weight gain are rather increased when the carbohydrate content is relatively low which could be explained by increased gluconeogenesis. However, it should be noted that comparison between studies is difficult as macronutrient composition and measurement techniques differed substantial. This is also shown in the large range in costs of weight gain of 23 to 54 MJ/kg with 'average/mixed diet' overfeeding.

#### Components of energy expenditure

The component of daily energy expenditure most affected by changes in body weight is the BMR [1], any adaptive changes in total energy expenditure are therefore likely to appear in this component. Several studies reported an increased BMR after overfeeding [4,19,22-29]. This increase is due to the energy cost of fat and fat-free mass gains as well as the costs of maintaining a larger body weight [1].

Another component, DIT, will increase due to the increased amount of food that has to be digested and absorbed. Yet, several studies did not find a significant increase in DIT, independent of dietary composition and duration of the experiment [22,28-30]. Others could explain significant increases in DIT solely by the increased amount of EI, as reflected by the percentage of the EI found in the DIT component being similar before and after overfeeding [27] or the response to a fixed meal being unaltered [25]. Pasquet et al. [26] reported a similar increase in DIT with long-term high-carbohydrate overfeeding compared to overfeeding with a typical western, mixed diet [22,28-30], but concluded that this increase included an adaptive component as the increase was even larger after adjusting for a reduction in physical activity.

The last component, AEE, is the most variable component of TEE between persons [31], and thus is most likely the main contributor to variation in weight gain during overfeeding. Indeed, several overfeeding experiments show that those subjects with the largest increase or decrease in AEE have respectively the lowest and highest weight gains [4,25]. But relatively large changes in AEE (as percentage of TEE) above increased costs of performing physical activity due to an increased body weight, might reflect behavioral changes rather than adaptive thermogenesis.

It should be noted that the division of energy expenditure into its components may induce over- or underestimations of the separate components. AEE is particularly hard to determine, as measurement errors in TEE, BMR and DIT are accumulated in AEE [2]. SMR might be confounded by DIT; the influence of a large evening meal has been shown to continue well into the night [32], which might confound measurement of BMR in the morning as well [4,19]. In addition, there is an interaction between DIT and physical activity both at high and low levels of activity [33,34], which will not only affect DIT but will also influence determination of the energy costs of physical activity [4].

### Energy storage

Energy cannot get lost; energy that is not expended will be stored. As the digestibility of foods is not affected by intake level or subject [4,35], energy storage during overfeeding can be calculated as the difference between energy intake and energy expenditure.

The macronutrient composition of the diet can influence energy storage. With carbohydrate overfeeding 75 to 85% of the excess energy was stored and the remaining expended, while with fat overfeeding 90 to 95% of the excess energy was stored, but there was no difference in fat storage after 14 d between the two diets fed isoenergetically to the same subjects [14]. Lammert et al. [20] also found similar fat storage on high-carbohydrate and high-fat overfeeding. Overfeeding mixed diets resulted in a large variation in energy storage. The percentage of the excess energy intake that is stored ranged between 60 to 90% [22,27,28,30,36]. This variation is at least partly due to limitations in the measurement of small (< 1 kg) changes in body composition.

The composition of the overfeeding-induced body weight gain is fairly constant over different studies. Between 60 to 67% of the weight gain comprises an increase in fat mass (FM); the remaining part is an increase in fat-free mass (FFM) of 33 to 40% [4,20,22,30]. The high storage capacity of the adipose tissue, together with the low costs of fat gain (6.3 MJ/kg) compared to high costs of depositing protein (29.4 MJ/kg) favor the deposition of fat compared to fat-free mass.

In addition, there are other ways to store excess energy as fat. The storage of body fat from dietary fat is the most energy efficient (~0.02 MJ per MJ ingested fat), but dietary protein and carbohydrate can also be stored as fat (~0.25 MJ per MJ ingested protein or carbohydrate) [10]. Though several overfeeding studies showed the presence of *de novo *lipogenesis during carbohydrate overfeeding [20,37-39], the storage of carbohydrate as fat through *de novo *lipogenesis is considered a quantitavely negligible process under normal conditions in humans.

### Evidence or no evidence for adaptive thermogenesis during overfeeding

Overfeeding studies that have not found evidence for adaptive thermogenesis mainly base their conclusions on the observation that there is no elevation in metabolic rate above obligatory costs, i.e. EE associated with an increased body size and tissue gain [4,14,22,30,36], an increased DIT due to the increased amount of food eaten [4,27], increased costs for the same body movements due to an increased body weight [4,27] and a body weight gain proportional to the total amount of excess energy consumed [23,24,28]. All studies show a large inter-individual variation in weight gain, but comparing metabolically efficient and inefficient subjects showed no differences in EE changes [19]. Although these overfeeding experiments fail to show adaptive changes in energy expenditure, this does not mean there is no adaptive thermogenesis. In most studies there is still a considerable proportion of excess energy intake that was not accounted for [22,30,36], which is probably due to errors in the methods and assumptions used. In addition, the study period might have been too short, while adaptive thermogenesis is involved in long-term energy balance regulation [40].

Other studies conclude that adaptive thermogenesis must be present during overfeeding, because weight gain is smaller than expected [21] and the theoretical cost of storing dietary fat is exceeded [41]. They show that thermogenesis did increase above obligatory costs [21,25,26], either in DIT [26] or in the EE associated with PA like fidgeting, sitting and standing, which is called non-exercise activity thermogenesis (NEAT) [25].

If adaptive thermogenesis is present and contributes to the etiology of obesity then it is likely that obesity-prone persons have a reduced capacity for adaptive thermogenesis compared to obesity-resistant persons. As the predisposition to obesity in humans is hard to define, if possible at all, one usually compares lean and overweight or obese subjects. Results suggest that the thermogenic response to fat is flexible in lean subjects but that subjects with familial obesity have a reduced response [29]. Although fat oxidation differs between lean and obese subjects on overfeeding [4,14], the thermogenic response of lean and obese subjects was not different [4,14,21,40], but, as overfeeding experiments are designed to result in weight gain, the number of overweight and obese subjects willing to participate is for obvious reasons often limited.

## Conclusion

In humans, evidence for adaptive thermogenesis as a mechanism to explain interindividual differences in weight gain on the same overfeeding regimen is still inconsistent. Though most studies did find increases in EE during overfeeding, these were mainly explained by the theoretical energy costs of weight gain and the maintenance of a larger body weight. Changes in EE above these obligatory costs are considered adaptive thermogenesis, but the magnitude is generally no more than a few percent and includes measurement errors, errors in assumptions made and small (day-to-day) differences in physical activity. In addition, results from different overfeeding studies are hard to pool as there are marked differences in macronutrient composition, measurement techniques and availability of data within the papers. The latter causes comparison between studies using one measure (i.e. the costs of weight gain, Table 1) to be rather crude as often assumptions regarding absolute excess energy intake had to be made. Moreover, individual variation is lost using the mean values. This makes the existence of adaptive thermogenesis hard to prove. However, there are large differences in thermogenesis and weight gain between subjects, independent of body weight. In search for evidence for adaptive thermogenesis, it would therefore be interesting to define obesity-prone and obesity-resistant persons based on their response to overfeeding and in general it seems desirable to report individual data as well as group statistics.

## Competing interests

The author(s) declare that they have no competing interests.

## Authors' contributions

AMCPJ drafted the manuscript. KRW provided constructive, critical review of the manuscript.
